# Relationship Between Person-Organization Fit and Teacher Burnout in Kindergarten: The Mediating Role of Job Satisfaction

**DOI:** 10.3389/fpsyt.2022.948934

**Published:** 2022-07-08

**Authors:** Lingling Zang, Yameng Chen

**Affiliations:** Faulty of Education, Henan University, Kaifeng, China

**Keywords:** teacher burnout, person-organization fit, job satisfaction, kindergarten teacher, mediation analysis

## Abstract

As an important organizational factor, person-organization fit in kindergartens may lead to teacher burnout when it is unfitted. In order to explore the influence mechanism of person-organization fit on teacher burnout in kindergartens, this study introduced the variable of job satisfaction to study the mediating effect of job satisfaction on the relationship between person-organization fit and teacher burnout in kindergartens. A total of 637 teachers from Henan, China, were surveyed by Person-Organization Fit Scale, Job Satisfaction Scale and Maslach Burnout Inventory. Results showed that person-organization fit, job satisfaction and teacher burnout were negatively correlated. Person-organization fit is positively correlated with job satisfaction. Job satisfaction partially mediated the relationship between person-organization fit and teacher burnout in kindergartens. In the end, the results of the relationship between person-organization fit, job satisfaction, and teacher burnout in Chinese kindergartens were discussed in this study.

## Introduction

At present, teacher burnout arouses widespread concern gradually (1, 2). Kindergarten teachers who work with children aged 3–6 have dual responsibility of childcare and education, which leads to heavy workload, great work pressure and prone to burnout (3, 4). Teacher burnout affects the quality of education and also leads to teachers' dimission (5, 6), which is not conducive to the stability of the teaching staff and the development of education. In China, kindergarten teachers are generally facing the contradiction between high work pressure and low salary. As a result, burnout is more likely to be happened onto kindergarten teachers and the dimission rate of kindergarten teachers is higher than that of other teachers (7, 8). Teacher burnout is a phenomenon with social and cultural context and has different causes and manifestations in different societies (2, 9). To explore the potential predictors of kindergarten teacher burnout under the context of Chinese society can not only reveal the influencing factors and mechanism of teacher burnout to reduce the occurrence of burnout, but also enrich the localization research on teacher burnout and provide the possibility of cross-cultural comparison for researches in different countries and regions.

Teacher burnout as a psychological syndrome in response to chronic excessive pressure on the job includes three dimensions, an overwhelming exhaustion, feelings of cynicism and detachment from the job, and a sense of ineffectiveness and lack of accomplishment (10). Previous studies on teacher burnout have focused on individual factors, including age (11), personality traits (12), etc. Little attention is paid to the impact of organizational factors. A growing body of research suggests that burnout is more of social phenomenon than an individual one (10, 13). Therefore, the influence of organizational factors, human factors and the interaction between them should be considered in relation to burnout (14), especially from the perspective of person-organization fit (POF). Current studies focus on the relationship between POF and work attitude, innovation behavior, and turnover intention (15–17), while there are few studies on person-organization fit and job burnout. At the same time, studies have shown that when individuals' perception is compatible with their organization, their job satisfaction will be improved (18, 19), and the improvement of job satisfaction can reduce burnout and turnover tendency (20). From what has been discussed above, it can be assumed POF, job satisfaction and teacher burnout are closely related. However, there are no studies on whether and how POF and job satisfaction affect teacher burnout in kindergartens. Therefore, this study focuses on the relationship between POF and teacher burnout in kindergartens and identified potential mechanisms (i.e., job satisfaction).

### Person-Organization Fit and Teacher Burnout in Kindergartens

Person-organization fit is defined as the compatibility between people and organizations, which is embodied in three aspects: value fit, demand-supply fit and demand-ability fit (21). According to the Job Demand-Resource model, individuals with sufficient resources will show more positive work attitudes and behaviors, while people may feel tired and disconnected in an environment with high work requirements and insufficient resources (22). Person-organization fit is an important support resource for teachers. From this perspective, when the degree of POF is high, it is easy to stimulate the positive power of teachers, while teachers with low degree of POF are more likely to feel job burnout (23). The positive correlation between POF and teacher burnout in primary and secondary school teachers has been proven (24), but this relationship has not been studied among kindergarten teachers. Meanwhile, research has found that compared with social factors and personal factors, organizational factors have the most influence on kindergarten teacher burnout (3). In kindergarten environment, organizational climate has a significant impact on teacher burnout, which is reflected in the correlation between a higher degree of supportive atmosphere and a lower degree of teacher stress and burnout (4). Thus, this study takes kindergarten teachers as samples to verify the relationship between POF and teacher burnout, so as to enrich research on teacher burnout in kindergartens.

### Job Satisfaction as a Mediator

Job satisfaction refers to the degree of positive emotional orientation generated by teachers when they consider various aspects related to job after they engaged in teaching (25). Job satisfaction is significantly affected by POF (26). Research show that teachers with higher POF are more satisfied with their campus and career respectively, that is, the higher the level of POF, the higher the degree of satisfaction (27). This proves that there is a strong correlation between POF and job satisfaction. Meanwhile, job satisfaction also has direct impact on teacher burnout, which has been confirmed by the previous study, namely, there is a significant correlation between job satisfaction and all dimensions of teacher burnout (19). Teachers with low happiness and satisfaction in school will have a higher risk of job burnout (28). Therefore, job satisfaction is not only affected by POF, but also affects teacher burnout. However, few studies have explored the mediating role of job satisfaction in the relationship between the two. Thus, this study aims to focus on kindergarten teachers and explore the relationship between POF and teacher burnout, and investigate the mediating role of job satisfaction in the relationship between POF and teacher burnout. Specifically, the research hypotheses are as follows:

H1: Person-organization fit, job satisfaction and teacher burnout in kindergartens are significantly correlated.
H2: Job satisfaction plays a mediating role in the relationship between person-organization fit and teacher burnout in kindergartens.

## Materials and Methods

### Participants and Procedures

All participants were kindergarten professional teachers from different cities in Henan Province, China. Prior to this survey, permission was granted from kindergartens and the informed consent is provided to all the participants. The questionnaires were distributed to teachers in 2021 and were collected within 1 week. A total of 700 questionnaires were sent out in this study, and 672 were retrieved, with a recovery rate of 96%. 637 questionnaires were obtained by eliminating invalid questionnaires from the 672 questionnaires collected, and the criteria for elimination included:(a) a regular pattern of responses; (b) missing data; (c) contradictory responses to relevant items (e.g., inconsistent responses to homogeneous items or consistent responses to opposing items). These 637 questionnaires formed the final sample. The demographic characteristics of the sample are shown in Table 1.

**Table 1:** Demographic information of the sample (*n* = 637).

**Characteristics**	***n*(%)**
**Area**	
Urban	497 (78.0)
Rural	140 (22.0)
**Gender**	
Male	13 (2.0)
Female	624 (98.0)
**Teaching experience**	
≤ 5 years	290 (45.5)
6–10 years	204 (32)
11–15 years	78 (12.2)
≥15 years	65 (10.2)

### Person-Organization Fit Scale

Person-organization fit scale compiled by Wang Ling was used to measure the matching degree of teachers and kindergartens (29). The scale includes three dimensions: values fit (e.g., my personal values are very similar to those of the kindergarten), demand-supply fit (e.g., the resources my job provides me are a good fit for the job I'm looking for), requirement-ability fit (e.g., job requirements are a good fit for my skills). This scale is adapted from the Person-Organization Fit Scale compiled by Cable and Derue (30), and is better adapted to Chinese kindergarten teachers with good reliability and validity. There are nine items in the scale and all items were scored on a 5-point Likert scale (1 = strongly disagree to 5 = strongly agree). There is no reverse scoring question in 9 items. The higher the score of the three dimensions, the higher the fit degree of teachers and kindergartens. In the study, Crobach‘s α is 0.94.Internal consistency is 0.91 for value fit, 0.88 for demand-supply fit, 0.90 for requirement-ability fit.

### Maslach Burnout Inventory

The Maslach Burnout Inventory (MBI), developed by Maslach and Jackson, contains three different versions: MBI-Human Service Survey (MBI-SS), MBI-Educators Survey (MBI-ES) and MBI-General Survey (MBI-GS) (10). The MBI-ES with good reliability and validity was used to investigate teacher burnout in this study, which is divided into three dimensions, namely emotional exhaustion (e.g., a day's work makes me tired), depersonalization (e.g., after this career, I became more cold to people than before) and low accomplishment (e.g., I have many meaningful achievements in my work). The scale showed good reliability and validity in Chinese samples (31–33). The MBI-ES consist of 22 items on a 7-point Likert scale ranging from 1 (strongly disagree) to 7 (strongly agree). Items 4, 7, 8, 9, 12, 14, 17, 18 and 19 will be scored in reverse. Composite measure of burnout was used in the study, that is, the lower the total score in the three dimensions, the lower the burnout degree. In our sample, Cronbach's α is 0.93. For internal consistency, emotional exhaustion was 0.89, depersonalization was 0.78, and low accomplishment was 0.87.

### Job Satisfaction Scale

Job satisfaction scale was used to evaluate the degree of satisfaction that teachers feel during their work. This study adopts the Kindergarten Teacher Job Satisfaction Questionnaire compiled by Wang Yongxian (34). The questionnaire has five dimensions, including job itself (e.g., I am satisfied with the current work schedule), working environment (e.g., I am satisfied with the interpersonal relationship between colleagues), advanced study and promotion (e.g., I think there are many opportunities for advanced study), work remuneration (e.g., I am satisfied with the current welfare system), principal (e.g., I think principal values teacher advice), a total of 30 questions. All items were obtained on 6-point Likert scale with 1 point for strongly disagree and 6 points for strongly agree. The higher the scores, the higher the teacher's job satisfaction. In the scale, Cronbach's α is 0.96. The internal consistency of five dimensions is 0.92, 0.84, 0.72, 0.91, 0.96 respectively, corresponding to job itself, working environment, advanced study and promotion, work remuneration and principal.

### Control Variables

This study controlled the potential impact of demographic variables (area, gender, and teaching experience) on dependent variables and mediating variables, as all of them may affect teacher burnout.

### Data Analysis

SPSS26.0 was used for descriptive statistics and correlation analysis of person-organization fit, teacher burnout and job satisfaction. The mediating effect of job satisfaction on person-organization fit and teacher burnout was examined by Bootstrap. The nonparametric percentile Bootstrap method of deviation correction is a method of repeated sampling from a sample, which means repeated sampling with a return from a given sample can produce multiple samples (35). The sampling size was set at 1,000 in the current study. Repeated sampling by many times, it can be obtained an estimate of the product of 1,000 coefficients and rank them from small to large by numerical value. The 2.5th and 97.5 percentile site constitute a confidence interval (CI) of 95%. If the CI does not include 0, the coefficient product is significant, in other word, the mediation effect is significant.

## Results

### The Relationship Between Person-Organization Fit, Job Satisfaction and Teacher Burnout

In this study, the means, standard deviations and correlations among three variables are presented in Table 2. The results showed that person-organization fit was negatively correlated with teacher burnout (*r* = −0.510, *P* < 0.001), among which, three dimensions of person-organization fit showed significant negative correlation with teacher burnout (value fit, *r* = −0.411, *P* < 0.001; demand-supply matching, *r* = −0.554, *P* < 0.001; requirement-ability fit, *r* = −0.404, *P* < 0.001), and the negative correlation between demand-supply fit and teacher burnout is more significant than the other two dimensions. Person-organization fit was positively correlated with job satisfaction (*r* = 0.667, *P* < 0.001), and job satisfaction was negatively correlated with teacher burnout (*r* = −0.618, *P* < 0.001). Thus, H1 was supported.

**Table 2:** Means, standard deviations and correlations among variables.

**Variable**	**Mean**	**SD**	**1**	**2**	**3**	**4**	**5**	**6**
POF	4.23	0.58	1					
TB	1.01	0.94	−0.51***	1				
JS	4.90	0.66	0.67***	−0.62***	1			
VF	4.26	0.63	0.89***	−0.41***	0.55***	1		
DSF	4.18	0.70	0.93***	−0.55***	0.68***	0.77***	1	
RAF	4.26	0.61	0.88***	−0.40***	0.56***	0.65***	0.74***	1

*POF, person-organization fit; TB, teacher burnout; JS, job satisfaction, VF, value fit; DSF, demand-supply fit; RAF, requirement-ability fit*.

***p < 0.001.

### Mediating Effect of Job Satisfaction

Table 3 shows the regression analysis results of the relationship between person-organization fit and teacher burnout, in which area, gender and teaching experience are the control variables. Results showed that person-organization fit positively predicted job satisfaction (β = 0.753, *p* < 0.001) and negatively predicted teacher burnout (β = −0.798, *p* < 0.001). When job satisfaction was included in the regression equation, the direct effect value of person-organization fit on teacher burnout is significantly reduced (β = −0.257, *p* < 0.001). These results indicate that job satisfaction has a significant mediating effect on the relationship between person-organization fit and teacher burnout.

**Table 3:** Regression analysis of the relationship among variables.

	**Model 1**	**Model 2**	**Model 3**	**Model 4**	**Model 5**
Constant term	4.243***	1.003**	2.539***	5.971***	6.691***
Control variables					
Gender	0.168	0.276*	−0.513*	−0.627**	−0.429*
Teaching experience	0.043	−0.004	−0.167***	−0.117***	−0.120***
Areas	0.143**	0.122**	−0.037	−0.014	0.073
Independent variables					
POF		0.753***		−0.798***	−0.257***
Mediate variables					
JS					−0.717***
R^2^	0.016	0.455	0.046	0.290	0.429
F	3.334***	132.005***	10.256***	64.593***	94.744***

*All variables in these models have been standardized. Model1 took satisfaction as dependent variable and only introduced controls variables. Model2 took satisfaction as the dependent variable, and introduced POF and control variables to conduct regression analysis. Model3 took teacher burnout as dependent variable and only introduced control variables. Model4 took teacher burnout as dependent variable, and introduced POF and control variables to conduct regression analysis. Model5 took teacher burnout as dependent variable and introduced POF, JS and control variables to make regression analysis. POF, person-organization fit; JS, job satisfaction*.

*p < 0.05, **p < 0.01, ***p < 0.001.

**Figure 1:**
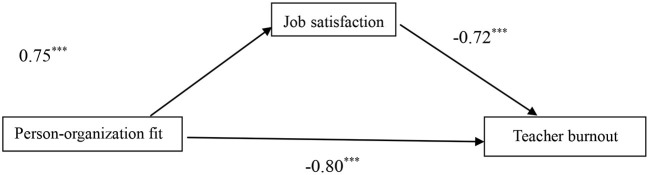
The mediation model of job satisfaction on the relationship between person-organization fit and teacher burnout. ***p < 0.001.

Bootstrap was used to further test the mediating effect. Results are showed in Table 4 and Figure 1. After controlling for demographic variables (area, gender and teaching experience), the total effect of person-organization fit on teacher burnout is −0.80 (SE=0.05, *t* = −14.73, *P* < 0.001, 95% CI [−0.90, −0.69]). The mediating effect of job satisfaction on person-organization fit and teacher burnout is −0.54 (SE = 0.06, 95% CI [−0.66, −0.43]), excluding 0.This proved that the mediating effect is significant, and job satisfaction plays a partial mediating role in the effect of person-organization fit on teacher burnout. Therefore, H2 was supported.

**Table 4:** Mediating effect of job satisfaction.

	**Effect**	**SE**	**t**	***p***	**LLCI**	**ULCI**
POF → TB(c)	−0.80	0.05	−14.73	0.0000	−0.90	−0.69
POF → JS(a)	0.75	0.03	22.58	0.0000	0.69	0.82
JS → TB(b)	−0.72	0.06	−12.38	0.0000	−0.83	−0.60
POF → TB(c')	−0.26	0.07	−3.94	0.0001	−0.39	−0.13
POF → JS → TB	−0.54	0.06			−0.66	−0.43

*C, the total effect of person-organization fit on teacher burnout; a, the effect of person-organization fit on job satisfaction; b, the effect of job satisfaction on teacher burnout; c‘, the direct effect of person-organization fit on teacher burnout when job satisfaction was included; LLCI, the lowest value of the confidence interval; ULCI, the highest value of the confidence interval; POF, person-organization fit; TB, teacher burnout; JS, job satisfaction*.

## Discussion

This study investigated the relationships among person-organization fit, teacher burnout and job satisfaction in kindergartens. Results showed that there was a significant negative correlation among three dimensions of person-organization fit and teacher burnout, confirming a previous study, which found that when individuals can form a good sustainable fit with the organizational environment, they will be actively engaged in work; On the contrary, individuals may experience burnout (36). In addition, the negative correlation between demand-supply fit and teacher burnout is stronger than the other two (value fit and requirement-ability fit). The result is consistent with the Job Demand-Resource Model. According to this model, compared with job resources, job demand is more significantly correlated with burnout, that is, individuals are more likely to suffer burnout when job fails to meet their needs (22). This indicates that compared with the fit of value and requirement-ability, teachers care more about whether their needs can be met in kindergartens. When teacher needs and organizational supply are unbalanced, individuals will suffer from burnout. As can be seen from Table 2, the score of demand-supply fit is the lowest compared with the other two dimensions, which may be largely related to the low salary and low social status of kindergarten teachers in China (8). The results of this study suggest that kindergarten managers should consider various needs of teachers, pay attention to the living state and quality of teachers, and provide targeted support for them to reduce burnout.

The current study found that person-organization fit in kindergartens is positively correlated with job satisfaction of teachers. This finding is consistent with previous studies. Teachers with a high degree of person-organization fit are more satisfied with their organizations and careers (27). A study on the relationship between person-organization fit and job satisfaction of young teachers in colleges and universities confirmed that all three dimensions of person-organization fit had a significant positive impact on teachers' job satisfaction (18). A study on person-organization fit and job-related happiness of kindergarten teachers also found that there was a significant positive correlation between person-organization fit and job-related happiness (37). These indicate that when individuals are well matched with their work requirements provided by the organization, they are more willing to make more contributions to the organization, and more satisfaction can be felt (26, 38, 39). Job satisfaction mainly comes from satisfying the needs to individuals (40, 41), and teachers' needs are usually obtained from the environment of kindergarten. Therefore, if there is a good interaction between teachers and kindergartens, the higher the fit between individuals and the organization, the higher the satisfaction will be.

This study also showed that there is a significant negative correlation between job satisfaction and teacher burnout. The finding is in consistence with a systematic review of longitudinal studies on determinants of teacher burnout,which found that job satisfaction was the most predictive factor (42). As a positive attitude related to job, job satisfaction can significantly negatively predict teacher burnout (43). Other studies also showed that the more satisfied teachers are with their work and salary, the more harmonious their interpersonal relationships are, and the less job burnout are experienced (44–46). A study of 564 in–service teachers in Turkey found a significant positive correlation between job satisfaction and teacher burnout. It also pointed out that “happiness” and “love” were significantly positively correlated with job satisfaction, and “sadness” and “fear” had significant predictive effects on teacher burnout (47). It can be concluded that teachers' higher job satisfaction can effectively reduce job burnout (48, 49). Therefore, to improve teachers' job satisfaction will help to reduce teacher burnout.

Finally, results confirm that job satisfaction plays a partially mediating role in the relationship between person-organization fit and teacher burnout. This fits nicely into social exchange theory. According to this theory, when individuals receive sufficient material and spiritual support from an organization, their job satisfaction will be improved, and in return, they will increase work involvement and reduce job burnout (50). Other studies have proven that when teachers perceive a high degree of fit with the organization, they will express a positive attitude toward work, and their perception of job burnout will be correspondingly weakened (51). Meanwhile, if teachers' job satisfaction is improved, their perceived burnout will be reduced and their enthusiasm to work will be improved (46, 52). Therefore, improving the degree of person-organization fit will improve job satisfaction and reduce mobility intention for teachers (53). The Conservation of Resources Theory proposes that individuals with more resources will not only do their best to maintain existing resources, but will also be better able to acquire new resources. Thus, individuals are also willing to invest resources in an organization, resulting in more positive experience evaluation and job satisfaction (54). In general, the more support and help that teachers received from an organization, the more satisfaction they will have with the organization, thus the work involvement will be increased and the burnout will be reduced (19, 55). These suggest that adjusting the job demands and job resources at the organization level will safeguard against daily stress and chronic burnout (56). Therefore, person-organization fit of kindergarten teachers not only directly affects job burnout, but also indirectly affects teacher burnout through job satisfaction. These results suggest that kindergarten managers can take positive measures in person-organization fit and job satisfaction to reduce teacher burnout.

### Practical Implications

Based on the social and cultural background of China, this study introduced person-organization fit for the first time and examined the effects of job satisfaction and person-organization fit on teacher burnout in kindergarten teachers, and found that improving person-organization fit and job satisfaction in kindergartens can significantly reduce teacher burnout. This result can provide practical implications for kindergarten managers and policy makers. On the one hand, teachers' needs should be considered from the perspective of person-organization fit, and the fit degree should be improved to enhance teachers' job satisfaction and reduce burnout. On the other hand, it is necessary to create a more friendly kindergarten environment, such as offering a transparent and fair system for promotion and continuous study, and a system of compensation to matching their efforts, so that teachers can focus more on teaching itself and reduce burnout.

## Limitations

There are limitations to this study that need to be stated. Firstly, this study only selected 637 kindergarten teachers from Henan Province for investigation. The sample size is not sufficient and the selected subjects are not broad enough, which may reduce the universality of the results. The sample size can be expanded to cover more provinces and regions in China in the future. Secondly, this study adopts a cross-sectional design, which is not conducive to explaining the causal relationship between variables. In the future, longitudinal research design or experimental research can be adopted to verify the impact of human-organization fit and job satisfaction on teacher burnout. Thirdly, the current study only examined the impact of person-organization fit on teacher burnout, but there are other organizational factors that affect teacher burnout, which can be further explored. Finally, the mediating model shows that the job satisfaction is partially mediated between person-organization fit and teacher burnout, suggesting that there are other mediating factors existed. Future studies can introduce other mediating variables, such as psychological capital and work-family balance, to better understand the impact of person-organization fit on teacher burnout, which can help alleviate teacher burnout.

## Conclusion

This study is the first to explore the relationships between person-organization fit, job satisfaction and teacher burnout. The results not only find the direct impact of person-organization fit on teacher burnout, but also confirm the mediating role of job satisfaction between the two. It enriches the existing research literature in understanding the relationship between person-organization fit, teacher burnout and job satisfaction of kindergarten teachers in the context of Chinese culture. In addition, other possible mediating factors between person-organization fit and teacher burnout and antecedent variables of teacher burnout deserve further exploration.

## Data Availability Statement

The original contributions presented in the study are included in the article/Supplementary Material, further inquiries can be directed to the corresponding author.

## Ethics Statement

The studies involving human participants were reviewed and approved by Faculty of Education, Henan University. The patients/participants provided their written informed consent to participate in this study.

## Author Contributions

LZ and YC: research idea and research design. YC: data collection and analysis. LZ: manuscript writing. All authors contributed to the article and approved the submitted version.

## Funding

The study was funded by Henan Province Key R&D and promotion project in 2021 (202102310570) and the Project of Teacher Education Curriculum Reform Research in Henan Province (2021-JSJYYB-008).

## Conflict of Interest

The authors declare that the research was conducted in the absence of any commercial or financial relationships that could be construed as a potential conflict of interest.

## Publisher's Note

All claims expressed in this article are solely those of the authors and do not necessarily represent those of their affiliated organizations, or those of the publisher, the editors and the reviewers. Any product that may be evaluated in this article, or claim that may be made by its manufacturer, is not guaranteed or endorsed by the publisher.
